# Gut Microbiota Profiling in Patients With HER2-Negative Metastatic Breast Cancer Receiving Metronomic Chemotherapy of Capecitabine Compared to Those Under Conventional Dosage

**DOI:** 10.3389/fonc.2020.00902

**Published:** 2020-07-07

**Authors:** Xiuwen Guan, Fei Ma, Xiaoying Sun, Chunxiao Li, Lixi Li, Fang Liang, Shaochuan Li, Zongbi Yi, Binliang Liu, Binghe Xu

**Affiliations:** ^1^Department of Medical Oncology, National Cancer Center/National Clinical Research Center for Cancer/Cancer Hospital, Chinese Academy of Medical Sciences and Peking Union Medical College, Beijing, China; ^2^State Key Laboratory of Molecular Oncology, Chinese Academy of Medical Sciences, Beijing, China; ^3^Department of Human Microbiome, Promegene Institute, Shenzhen, China

**Keywords:** breast cancer, metronomic chemotherapy, gut microbiota, capecitabine, maintenance chemotherapy

## Abstract

**Purpose:** Low-dose metronomic chemotherapy can achieve disease control with reduced toxicity compared to conventional chemotherapy in maximum tolerated dose. Characterizing the gut microbiota of cancer patients under different dosage regimens may describe a new role of gut microbiota associated with drug efficacy. Therefore, we evaluated the composition and the function of gut microbiome associated with metronomic capecitabine compared to conventional dosage.

**Methods:** The fecal samples of HER2-negative metastatic breast cancer patients treated with capecitabine as maintenance chemotherapy were collected and analyzed by 16S ribosome RNA gene sequencing.

**Results:** A total of 15 patients treated with metronomic capecitabine were compared to 16 patients under a conventional dose. The unweighted-unifrac index of the metronomic group was statistically significantly lower than that of the routine group (*P* = 0.025). Besides that, the Bray–Curtis distance-based redundancy analysis illustrated that the microbial genera between the two groups can be separated partly. Nine Kyoto Encyclopedia of Genes and Genomes (KEGG) modules were enriched in the metronomic group, while no KEGG modules were significantly enriched in the routine group. Moreover, univariate and multivariate analyses suggested that the median progression-free survival (PFS) was significantly shorter in patients with the gut microbial composition of *Slackia* (9.2 vs. 32.7 months, *P* = 0.004), while the patients with *Blautia obeum* had a significantly prolonged PFS than those without (32.7 vs. 12.9 months, *P* = 0.013).

**Conclusions:** The proof-of-principle study suggested that the gut microbiota of patients receiving metronomic chemotherapy was different in terms of diversity, composition, and function from those under conventional chemotherapy, and the presence of specific bacterial species may act as microbial markers associated with drug resistance monitoring and prognostic evaluation.

## Introduction

Capecitabine, an oral prodrug of fluorouracil, has been proven effective in metastatic breast cancer and is widely used as first-line chemotherapy in patients resistant to anthracycline, taxane, or both (1–4). Besides that, in HER2-negative breast cancer patients who have residual invasive disease on pathological testing after a standard neoadjuvant chemotherapy containing anthracycline and taxane, the addition of adjuvant capecitabine therapy is also proven to prolong disease-free survival and overall survival (5). Due to the fact that the standard clinical practice for metastatic breast cancer is to continue first-line chemotherapy until progression or intolerable toxicity (2, 6), the adverse effects caused by conventional chemotherapy, such as hand–foot syndrome, nausea, diarrhea, and hematologic adverse events, may lead to dose reduction and cessation of chemotherapy prior to disease progression (1, 3, 4, 7, 8).

Metronomic chemotherapy refers to chemotherapy in low (1/10–1/3 of the maximum tolerated dose), minimally toxic doses on a frequent schedule of administration, which has evolved from preclinical studies since over a decade ago and has been investigated in several clinical trials in different tumor types (9–12). In metastatic breast cancer, treatment is intended to prevent tumor progression for a relatively extended period of time; thus, low-dose metronomic administration of chemotherapy may be an ideal choice for maintenance treatment. *Via* lower doses and more frequent administration, metronomic chemotherapy may reduce the toxic effects and achieve disease control by preventing rapid vascular regrowth during therapy breaks (13–15). Capecitabine is one of the ideal agents in a metronomic schedule for its antiangiogenic activity resulting from the metronomic dosage (9).

In recent years, the correlation between the gut microbiota and chemotherapy has attracted much attention. Many pharmaceutical agents influence the composition of intestinal microbiota, while gut microorganisms may also modulate the efficacy and the toxicity of drugs (16–18). Evidence showed that the efficacy of chemotherapeutic agents may also depend on the innate and the adaptive immune responses mediated by microorganisms (19, 20). A previous study explored the role of microbes in modulating the effect of 5-FU and other fluoropyrimidines on *Caenorhabditis elegans* and found that bacteria are key determinants of fluoropyrimidine efficacy on host metabolism (21). However, no previous studies addressed the relationship between the different dosage regimens of anti-cancer drugs and the gut microbiota. In this study, we used 16S rRNA gene sequencing to explore the gut microbiota of fecal samples from patients with HER2-negative metastatic breast cancer to compare the composition of the gut microbiota in patients who receive metronomic capecitabine as maintenance treatment with those taking the conventional dosage. We identified the bacterial taxa associated with the two different dose-intensity regimens and revealed the differences of function between the two bacterial taxa, which may provide potential basis for clinical practice.

## Materials and Methods

### Study Design and Fecal Sample Collection

This was a prospectively designed retrospective translational medicine study. The objective was to compare the distribution and the functional characterization of gut microbiota profiling in patients who receive metronomic capecitabine as maintenance treatment with those taking the conventional dosage. The study protocol was approved by the Institutional Review Board of China National Cancer Center, and all patients provided informed consent before enrollment in this study. Main eligibility included (1) confirmed histologic/cytologic diagnosis of HER2-negative metastatic breast cancer, (2) receiving first-line chemotherapy with docetaxel plus capecitabine for six cycles and then randomized to receive the maintenance chemotherapy with capecitabine of either conventional dosage regimens or metronomic dosage regimens, (3) current maintenance chemotherapy with single-agent capecitabine for more than 1 month, (4) with measurable lesions defined by the revised Response Evaluation Criteria in Solid Tumors guidelines version 1.1 (RECIST 1.1), (5) Eastern Cooperative Oncology Group performance status <2, and (6) adequate hematologic, hepatic, and renal function. The patients were excluded if they had ulcerative colitis, Crohn's disease, malabsorption syndrome, or a disease significantly affecting gastro-intestinal function that could affect the absorption of oral capecitabine. Besides that, the patients with a clinically significant medical illness, including severe/uncontrolled hypertension, diabetes, or heart disease, were also excluded.

Fecal samples were collected when the patients were receiving the maintenance chemotherapy with single-agent capecitabine in either conventional regimens (1,000–1,250 mg/m^2^ twice daily, given on days 1–14 every 3 weeks) or metronomic regimens (500 mg, thrice daily) for at least 1 month between November 2017 and February 2019. The last follow-up visit was conducted in June 2019.

All the fecal samples collected during the maintenance chemotherapy of capecitabine as prospectively designed were stored in sealed plastic containers and transferred to be frozen at −80°C within 30 min. A retrospective analysis on gut microbiota profiling was conducted when the specimen collection was completed.

### DNA Extraction and 16S Ribosome RNA V4 Region Sequencing

DNA extraction was conducted according to the MOBIO PowerSoil® DNA Isolation Kit 12888-100 protocol, and DNA was stored at −80°C in Tris-ethylenediaminetetraacetic acid buffer solution before use. To enable the amplification of the V4 region of the 16S rRNA gene and add barcode sequences, unique fusion primers were designed based on the universal primer set, 515F (5′-GTGYCAGCMGCCGCGGTAA-3′) and 806R (5′-GGACTACNVGGGTWTCTAAT-3′), along with barcode sequences. The PCR mixtures contained 1 μl of each forward and reverse primer (10 μM), 1 μl of template DNA, 4 μl of dNTPs (2.5 mM), 5 μl of 10× EasyPfu Buffer, 1 μl of Easy Pfu DNA Polymerase (2.5 U/μl), and 1 μl of double-distilled water in a 50-μl reaction volume. Thermal cycling consisted of an initial denaturation step at 95°C for 5 min, followed by 30 cycles of denaturation at 94°C for 30 s, annealing at 60°C for 30 s, and extension at 72°C for 40 s, with a final extension step at 72°C for 4 min. Amplicons from each sample were run on agarose gel. The expected band size for 515f-806r is ~300–350 bp. The amplicons were quantified with Quant-iT PicoGreen dsDNA Assay Kit (ThermoFisher/Invitrogen cat. no. P11496; follow the manufacturer's instructions). The amplicon library for high-throughput sequencing on the Illumina MiSeq platform was combined in an equal amount and subsequently quantified (KAPA Library Quantification Kit KK4824) according to the manufacturer's instructions.

### Profiling of 16S rRNA Gene Sequencing Data and Bioinformatic Analyses

Using the Quantitative Insights into Microbial Ecology (QIIME2, https://qiime2.org/) platform and the standard tools/plugins provided by QIIME2 (22), the raw sequences were processed to concatenate reads into tags according to the overlapping relationship; then, reads belonging to each sample were separated with barcodes and the low-quality reads were removed. The processed tags were clustered into operational taxonomic units (OTUs) at the commonly used 97% similarity threshold. The OTUs were assigned to taxa by matching to the Greengenes database (Release 13.8). A phylogenetic tree of representative sequences was built. Alpha and beta diversity analyses were performed. The distances were calculated with R (3.3.1, flexmix package).

### Statistical Analyses

Statistical analyses were performed using SPSS 22.0 software, GraphPad Prism 8.0, or R (3.3.1). In the analysis, *P*-values < 0.05 were considered as statistically significant. The comparison of the clinicopathological characteristics between the metronomic group and the routine group was determined by using chi-square test or Fisher's test. The difference of gut microbiota composition between different capecitabine dosage regimens in OTU counts, alpha diversity indexes, and beta diversity indexes was calculated by nonparametric Mann–Whitney or Kruskal–Wallis tests. Pearson test was conducted to investigate the association between gut microbial composition and progression-free survival (PFS), which was verified by Kaplan–Meier analysis and Cox proportional hazards regression analysis.

## Results

### Study Cohort and Sequencing Data

A total of 31 patients hospitalized in China National Cancer Center, including 15 metastatic breast cancer patients treated with a metronomic dose of capecitabine and 16 patients treated with conventional dose, were enrolled. Their clinicopathologic characteristics are summarized in Table 1. The median age of the 31 patients was 50 (range, 36–66) years and 25 (80.6%) of them were hormone receptor positive. No significant difference was observed in age, menstrual status, hormone receptor status, position of metastatic site, disease-free survival, previous endocrinotherapy, and adverse events between patients treated with different dose-intensity dosage regimens of capecitabine. The median PFS of all patients was 16.9 months (range, 5.5–56.6 months). No significant survival difference was found between the metronomic group and the routine group (median PFS 32.7 vs. 16.9 months; average PFS 25.4 vs. 30.9 months; *P* = 0.703). All the samples were collected during the maintenance chemotherapy with single-agent capecitabine, and no significant difference was observed in the time from the start of the maintenance chemotherapy with capecitabine to the timepoint of sampling between the metronomic group and the routine group (χ^2^ = 0.059, *P* = 0.971).

**Table 1:** Clinicopathological characteristics of the metronomic group and the routine group.

	**Metronomic group (*n* = 15)**	**Routine group (*n* = 16)**	**χ^**2**^**	***P*-value**
Age			0.027	0.870
<50	7 (46.7%)	7 (43.8%)		
≥50	8 (53.3%)	9 (56.2%)		
Menstrual status			0.511	0.774
Premenopausal	7 (46.6%)	7 (43.7%)		
Perimenopausal	4 (26.7%)	6 (37.5%)		
Postmenopausal	4 (26.7%)	3 (18.8%)		
HR status				1.000
Positive	12 (80.0%)	13 (81.3%)		
Negative	3 (20.0%)	3 (18.8%)		
Disease-free survival				1.000
<24 months	5 (33.3%)	5 (31.3%)		
≥24 months	10 (66.7%)	11 (68.8%)		
Position of metastatic site			0.027	0.870
Non-visceral	7 (46.7%)	7 (43.8%)		
Visceral	8 (53.3%)	9 (56.3%)		
Previous endocrinotherapy (after confirmed relapse)			3.029	0.220
None	13 (86.7%)	10 (62.5%)		
1st line	2 (13.3%)	4 (25.0%)		
2nd line or more	0	2 (12.5%)		
Adverse events			2.602	0.626
Hand–foot syndrome	4 (26.7%)	5 (31.3%)		
Hematologic adverse event	1 (6.7%)	2 (12.5%)		
Gastrointestinal disorders	2 (13.3%)	4 (25.0%)		
Fatigue	0	1 (6.3%)		
None	9 (60.0%)	6 (37.5%)		
Time from the start of maintenance capecitabine to the timepoint of sampling			0.059	0.971
1 to 3 months	6 (40.0%)	6 (37.5%)		
3 months to 1 year	4 (26.7%)	4 (25.0%)		
More than 1 year	5 (33.3%)	6 (37.5%)		

We obtained 1,856,628 high-quality data (59,891 ± 10,883 sequences per sample; minimum, 37,636) on the participants' gut samples by using bacterial 16S rRNA gene sequencing of the V4 variable region. A total of 965 OTUs were identified and functionally labeled based on QIIME2, an open source, which has been described in “**Materials and Methods**.”

### Reduced Diversity in the Metronomic Group

**Figure 1:**
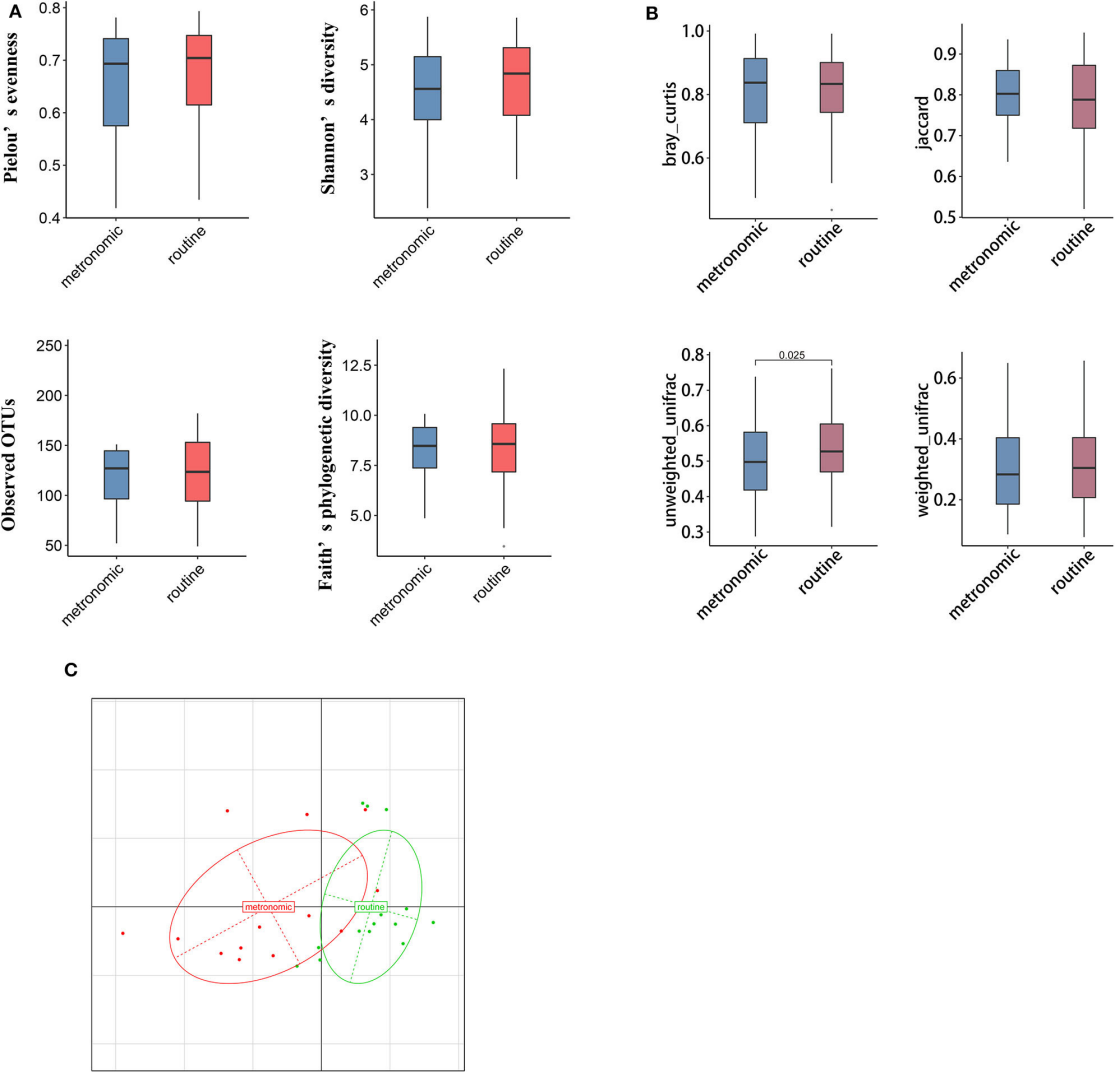
Comparison of the diversity of gut microbiota composition between the metronomic group and the routine group. **(A)** Comparison of the gut microbiota composition in α diversity between the metronomic group and the routine group. **(B)** Comparison of the gut microbiota composition in β diversity between the metronomic group and the routine group. **(C)** Bray–Curtis distance-based redundancy analysis between the metronomic group and the routine group.

We used α and β diversity to evaluate the intersample and intrasample relationship of the gut microbiota of all participants. Except for the Observed_OTUs index, the other three indices (including Shannon diversity index, Pielou's evenness, and Faith's phylogenetic diversity) were lower in the metronomic group when compared with those of the routine group (Figure 1A), while the differences of α diversity between the two groups were not statistically significant. Differently, the unweighted-unifrac index of the metronomic group was significantly lower than that of the routine group (*P* = 0.025) (Figure 1B). Furthermore, the Bray–Curtis distance-based redundancy analysis (dbRDA) illustrated that the microbial genera between the two groups can be separated partly, while the genera *Bacteroides, Clostridium2*, and *Megamonas* represented the major contributors (Figure 1C).

### Associations of Gut Microbiota and Capecitabine Dosage Regimen

**Figure 2:**
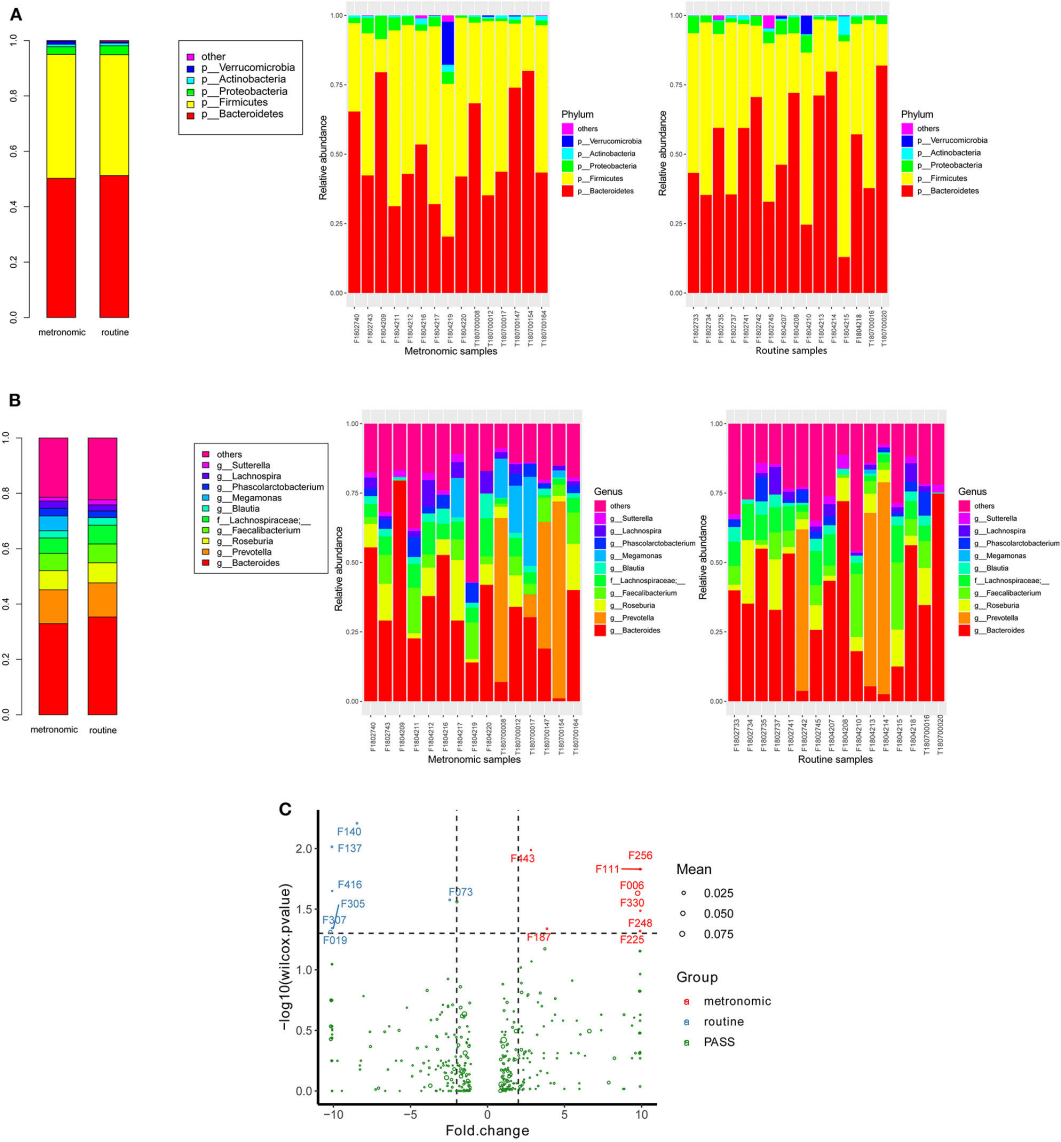
Comparison of the distribution of gut microbiota between the metronomic group and the routine group. **(A)** Distribution of the gut microbiota composition in the metronomic group and the routine group at the phylum level. **(B)** Distribution of the gut microbiota composition in the metronomic group and the routine group at the genus level. **(C)** Comparison of the gut microbiota composition between the metronomic group and the routine group at the level of operational taxonomic units.

The microbial signatures of the different dosage regimens of capecitabine were investigated by the gut microbial composition of the metronomic group and the routine group. At the phylum level, the patients of the two dosage regimens have a similar microbial composition in the predominant phyla, with *Bacteroidetes, Firmicutes, Proteobacteria*, and *Actinobacteria* consisting more than 95% relative abundance in both two groups (Figure 2A), while *Cyanobacteria* was significantly depleted in the metronomic group. In addition, it is notable that *Chloroplast* was significantly more abundant in the routine group at the class level, and *Streptophyta* was significantly enriched in the routine group at the order level, but at the family level, *o_Streptophyta* was still significantly more abundant in the routine group, while *Veillonellaceae* was found to be less abundant in the routine group (Table 2).

**Table 2:** Comparison of the distribution of gut microbiota between the metronomic group and the routine group at different levels.

**Taxa**		**Metronomic**	**Routine**	***P*-value**
Phylum	*Bacteroidetes*	50.245	51.252	Ns
	*Firmicutes*	44.719	43.599	Ns
	*Proteobacteria*	2.780	3.267	Ns
	*Actinobacteria*	0.899	0.938	Ns
	*Cyanobacteria*	0.008	0.298	0.029
Class	*Bacteroidia*	50.245	51.252	Ns
	*Clostridia*	43.830	42.472	Ns
	*Betaproteobacteria*	1.360	1.838	Ns
	*Gammaproteobacteria*	1.029	0.764	Ns
	*Chloroplast*	0.003	0.021	0.049
Order	*Bacteroidales*	50.245	51.252	Ns
	*Clostridiales*	43.827	42.462	Ns
	*Burkholderiales*	1.360	1.838	Ns
	*Enterobacteriales*	0.989	0.736	Ns
	*Streptophyta*	0.003	0.021	0.049
Family	*Bacteroidaceae*	32.916	35.399	Ns
	*Lachnospiraceae*	22.171	26.103	Ns
	*Prevotellaceae*	12.241	12.316	Ns
	*Ruminococcaceae*	10.978	10.906	Ns
	*Veillonellaceae*	9.009	3.218	0.014
	*o_Streptophyta*	0.003	0.021	0.049
Genus	*Bacteroides*	32.916	35.335	Ns
	*Prevotella*	12.241	12.316	Ns
	*Roseburia*	6.937	7.269	Ns
	*Faecalibacterium*	6.211	6.777	Ns
	*f_Lachnospiraceae*	5.563	6.806	Ns
	*Blautia*	2.622	2.689	0.036
	*Megamonas*	5.329	0.090	0.023
	*Phascolarctobacteriumf*	2.773	2.336	Ns
	*f_Mogibacteriaceae*	0.024	0.006	0.042
	*o_Streptophyta*	0.003	0.021	0.049

At the genus level, the abundance ranks in the top five genera in both two groups were *Bacteroides, Prevotella, Roseburia, Faecalibacterium*, and *f_Lachnospiraceae*, which contributed 32.9, 12.2, 6.9, 6.2, and 5.6% of the metronomic group and 35.3, 12.3, 7.3, 6.8, and 6.8% of the routine group, respectively. It is worth noting that the relative abundance of all the five genera in the routine group was slightly higher than that in the metronomic group. In addition, *Megamonas* and *f_Mogibacteriaceae* were significantly enriched in the metronomic group, while *Blautia* and *o_Streptophyta* were depleted in the metronomic group (Figure 2B, Table 2).

A total of 1,029 bacterial OTUs were found in the present study, and 668 OTUs were detected in the metronomic group, while 665 OTUs were noted in the routine group. Moreover, seven OTUs, including F140 (*f_Lachnospiraceae*), F137 (*g_Roseburia*), F416 (*f_Lachnospiraceae*), F305 (*f_Lachnospiraceae*), F307 (*f_Lachnospiraceae*), F019 (*s_Bacteroides plebeius*), and F073 (*g_Lachnospira*), were identified to be more abundant in the routine group, while another eight OTUs, such as F443 (*g_Oscillospira*), F256 (*f_Ruminococcaceae*), F111 (*f_Enterobacteriaceae*), F006 (*g_Megamonas*), F330 (*g_Faecalibacterium*), F248 (*f_Lachnospiraceae*), F187 (*g_Oscillospira*), and F225 (*f_Barnesiellaceae*), were enriched in the metronomic group (Figure 2C, Table S1).

### Functional Characterization of the Gut Microbiota

**Figure 3:**
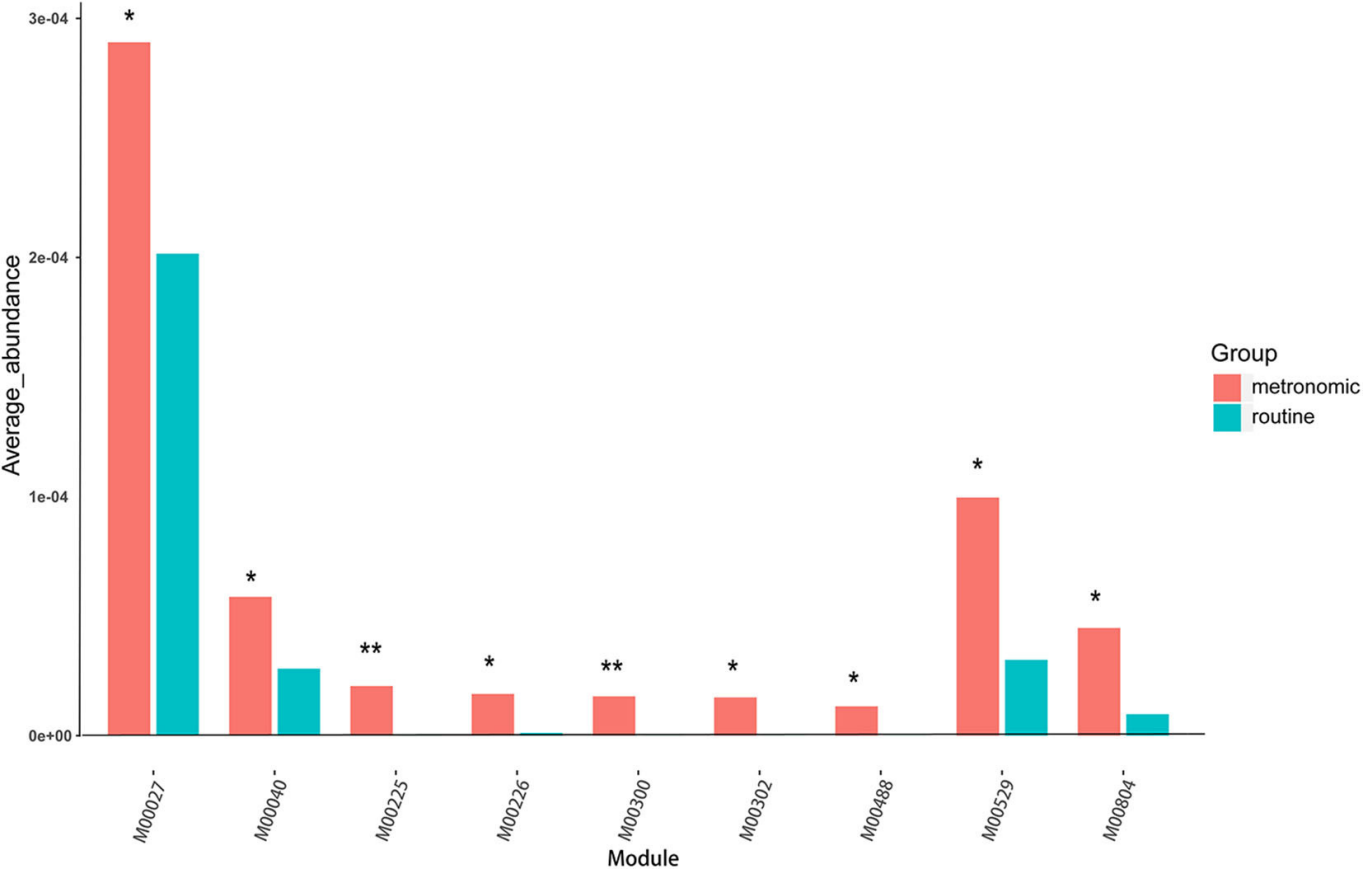
Comparison of the functional characterization of gut microbiota between the metronomic group and the routine group. *P < 0.05, **P < 0.01.

To infer the functional capacity of the gut microbiota of the patients in the metronomic group and the routine group, we utilized PICRUSt analysis based on their microbial community profiles. Finally, the relative abundance of 385 Kyoto Encyclopedia of Genes and Genomes (KEGG) modules was quantified after abundance filtering at an average relative abundance threshold of 0.001%. Nine KEGG modules were enriched in the metronomic group, corresponding to M00529 (denitrification), M00804 (complete nitrification), M00040 (tyrosine biosynthesis), M00027 (gamma-aminobutyrate shunt), M00300 (putrescine transport system), M00302 (2-aminoethylphosphonate transport system), M00225 (lysine/arginine/ornithine transport system), M00226 (histidine transport system), and M00488 (DcuS-DcuR) (C4-dicarboxylate metabolism) two-component regulatory systems, whereas no KEGG module was significantly enriched in the routine group (Figure 3).

### Association Between Gut Microbiota and Progression-Free Survival

We compared the microbial genera and OTUs of all the 31 breast cancer patients to investigate inter-associations between gut microbial composition and PFS and objective response. At the genus level, PFS was positively associated with *Veillonella* (ρ = 0.42), *Adlercreutzia* (ρ = 0.35), and *Akkermansia* (ρ = 0.31) and negatively associated with *Holdemania* (ρ = −0.41), *Paraprevotella* (ρ = −0.40), and *Slackia* (ρ = −0.39). In addition, the analysis results based on OTU level show that F076 (*s_Blautia obeum*, ρ = 0.57), F031 (*g_Blautia*, ρ = 0.50), F720 (*g_Holdemania*, ρ = −0.44), F036 (*s_Prevotella copri*, ρ = −0.43), and F547 (*g_Megamonas*, ρ = −0.43) were the top five OTUs for relevance (without distinguishing positive and negative). It is worth noting that the correlation between PFS and F256 (*f_Ruminococcaceae*), which was significantly different between the routine group and the metronomic group, reached −0.39.

**Figure 4:**
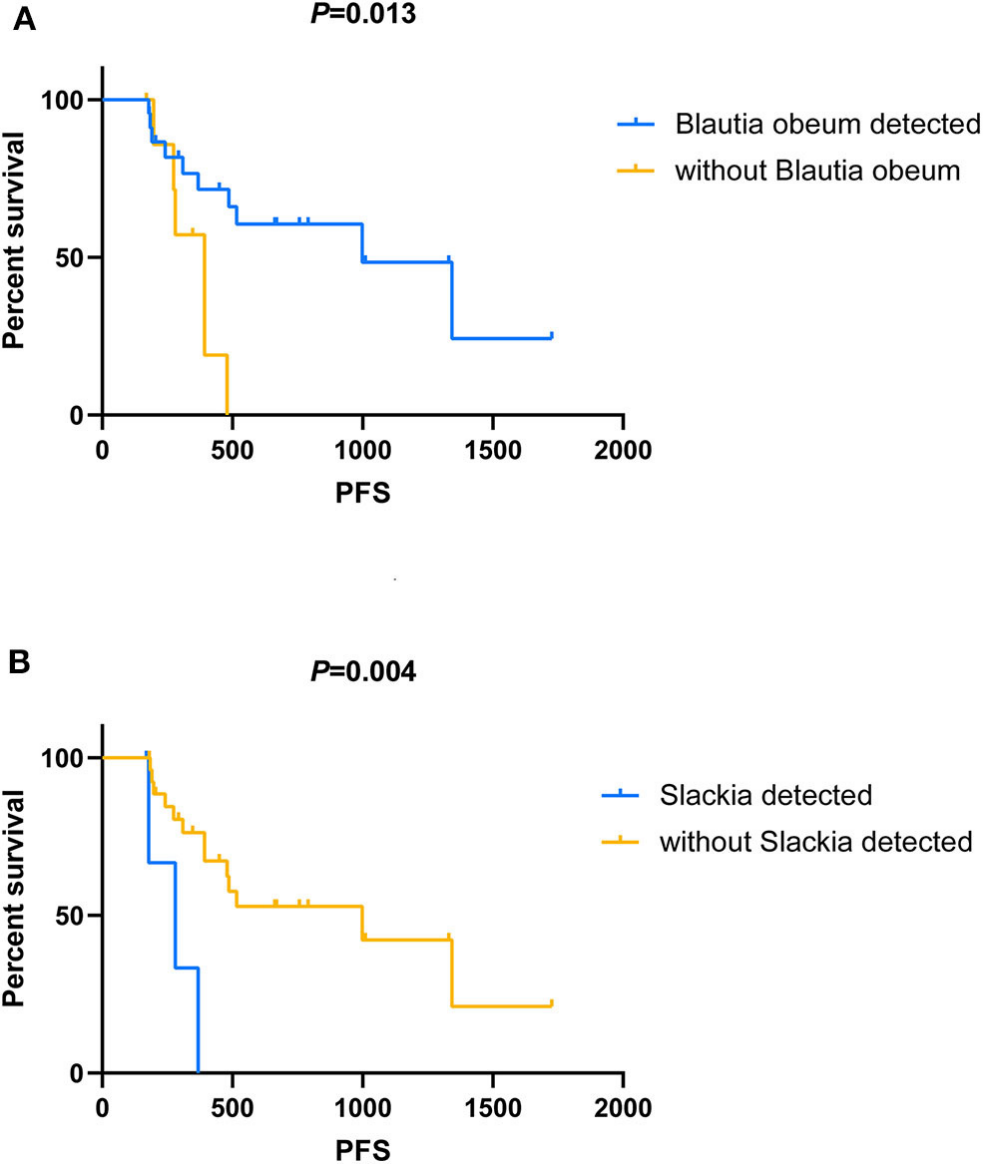
Kaplan–Meier analysis in patients with HER2-negative metastatic breast cancer who receive capecitabine as maintenance treatment. **(A)** Kaplan–Meier analysis-based estimation of progression-free survival (PFS) probabilities upon comparing the patients with the presence of *Blautia obeum* to those without. **(B)** Kaplan–Meier analysis-based estimation of PFS probabilities upon comparing the patients with the presence of *Slackia* to those without.

Among all the above-mentioned microbial genera and OTUs, the median PFS was significantly shorter in the patients with the gut microbial composition of *Slackia* (9.2 vs. 32.7 months, *P* = 0.004, Figure 4B), while the patients with *s_Blautia obeum* had a significantly prolonged PFS than those without (32.7 vs. 12.9 months, *P* = 0.013, Figure 4A). In the multivariate analysis, the presence of *Slackia* and *s_Blautia obeum* remained as the only significantly independently predictive factors associated with PFS, with the adjusted hazard ratios of 0.201 (95% CI: 0.048–0.837, *P* = 0.028) and 3.405 (95% CI: 1.045–11.093, *P* = 0.042; details of the other clinical indexes in the multivariate analysis are shown in Table S2). However, no single microbial genus and OUT was observed to be significantly correlated with objective response difference.

## Discussion

As for breast cancer, several previous studies investigated the association between gut microbiota and the development and the dissemination of breast cancer *via* regulation of inflammation, immunity, and metabolism (23–26). However, there was scarcely any evidence on the composition of gut microbiota in HER2-negative metastatic breast cancer patients who receive different dosage regimens of chemotherapy. To our knowledge, this study is the first attempt to characterize the stool microbial profile of fecal samples from metastatic breast cancer patients who were treated with a metronomic dose of capecitabine and compared with a similar population who received a conventional dose. Our study suggested that the gut microbiota of the patients who underwent metronomic chemotherapy was different in composition and function from those who receive conventional chemotherapy, and reduced diversity was observed in the metronomic chemotherapy cohort compared to the conventional dosage cohort. Additionally, our results indicated the presence of *s_Blautia obeum* and *Slackia* to have relatively exhibited a significantly positive and negative predictive association with PFS in the metastatic breast cancer patients when receiving the treatment of capecitabine.

In our study, although no significant difference was observed in α diversity between the two groups, the unweighted-unifrac index of the metronomic group was statistically lower than that of the routine group. Some previous studies showed that decreased microbial diversity of the gut always seems to be associated with disease or unhealthy conditions (27). The results are more consistent especially in gut diseases such as Crohn's disease (28), ulcerative colitis (29), and colorectal cancer (30). However, the observational study by Zhu et al. (31) suggested that the diversity of gut microbiota was higher in postmenopausal breast cancer patients than in healthy controls. In addition, in a Chinese cohort of advanced non-small cell lung cancer patients receiving the treatment of anti-programmed death 1 (PD-1) blockade, patients with better clinical responses exhibited higher gut microbiome diversity (32). Although some genetic or environmental factors which may affect the gut microbiota are not fully controlled in our research, we captured the visible separation of gut microbiota from samples with different capecitabine dosage regimens by dbRDA analysis based on the Bray–Curtis distance. In short, differences in α-diversity and β-diversity may suggest the possible changes of gut microbiota that are associated to dosage regimens.

*Bacteroidetes, Firmicutes*, and *Proteobacteria* were the predominant phyla in the present study, while a former study suggested that the dominant taxa of typical healthy adults at the phylum level were *Firmicutes, Bacteroidetes*, and *Actinobacteria* (33). Although different from the typical healthy adults, both *Bacteroidetes* and *Firmicutes* occupied a very crucial position in gut microbiota. This is not a coincidence for many studies have confirmed that *Bacteroides* and *Firmicutes* dominate the gut microbiota of adults (34–37). In a study of 31 female patients with early-stage breast cancer, *Firmicutes* and *Bacteroidetes* phyla were also the most numerous fecal microbiotas (23, 38). Furthermore, a study concentrated on the influence of anti-cancer treatment in colorectal cancer patients indicated that *Cyanobacteria* was a potential biomarker for colorectal cancer patients after chemotherapy (39). While *Cyanobacteria* appeared in both groups in our study, the relative abundance in the metronomic group was lower than in the routine group. These studies enhance the hypothesis that variations of the gut microbiota are associated with anti-cancer drug.

In addition, *Roseburia* and *Faecalibacterium* were the predominant genera in both groups. Meanwhile, the relative abundance of *s_Faecalibacterium prausnitzii* and *s_Roseburia faecis* at the species level was relatively high. It is easy to find that both *Roseburia* and *Faecalibacterium* are considered members of the human gut that produce butyrate (40, 41). Existing research shows that butyrate-producing species were closely related to human health. In ulcerative colitis, both *F. prausnitzii* and *Roseburia hominis* decreased in patients and is one of the evidences of dysbiosis in patients with ulcerative colitis (42). Moreover, we also found that *F. prausnitzii* and *Roseburia* spp. still play an important role in the progress of chronic kidney disease (43). These studies suggested that the relative abundance of butyrate-producing species may be an indicator for evaluating dosage regimen.

Many pharmaceutical agents influence the composition of intestinal microbiota, while gut microorganisms may modulate the efficacy and the toxicity of drugs as well (17, 26). In our study, both univariate and multivariate analyses showing significantly positive and negative predictive correlation with PFS were observed in *s_Blautia obeum* and *Slackia* relatively in the treatment of capecitabine for metastatic breast cancer patients. Although no previous study investigated the predictive value of the gut microbiota for drug resistance monitoring and prognostic evaluation in breast cancer, some evidence on these two types of microbes showed the similar predictive tendency in the association with gastrointestinal tumors. *Blautia obeum* was one of the gut microbes involved in the transformation of carcinogen heterocyclic amines, and the reduced abundance of the taxa may increase heterocyclic amine-induced colorectal cancer risk (44). *Slaxkia* in the human gut was reported by several studies to be involved in the onset of colorectal cancer, which may be used as a microbial biomarker in colorectal cancer for prevention, diagnosis, prognosis, and/or therapeutics (45, 46). Besides the intestinal tract, Coker et al. (47) also observed that *Slaxkia* in gastric mucosa showed an increasing correlation with disease progression in gastric cancer patients. Our study provided potential biomarkers of drug resistance monitoring and prognostic evaluation for patients with metastatic breast cancer, which may contribute to the optimization of existing chemotherapeutic protocols.

One of the limitations of our study was the limited sample size in both groups. No basal stool samples obtained before treatment also limited the ability of this proof-of-principle study to describe the longitudinal influence of chemotherapy on gut microbiota, which may fail to illuminate whether the difference in the distribution of gut microbiota was influenced by chemotherapy or it was preexisting. Lack of inter-individual heterogeneity matching, such as body mass index, diet, lifestyle, and other factors which may affect the gut microbiota, could also be a limiting factor in this study. Another limitation was that the differences observed in the intestinal microbiota are not an absolute criterion but a reference for patients to choose the best dosage regimen. Thus, future researches with a larger cohort from breast cancer patients will be needed to promote the understanding of the relationship between gut microbiome and capecitabine dosage regimen. Besides that, testing specific microbiota in direct experimental studies (e.g., animal model studies) is also beneficial to show the causality between the gut microbiome and capecitabine dosage regimen.

This proof-of-principle study indicated that different dose-intensity regimens of the same chemotherapeutic agent could make a difference in the gut microbiota profile of metastatic breast cancer patients, including diversity, composition, and functional structure. The presence of specific bacterial species may act as microbial biomarkers for the evaluation of treatment response and prognosis. These findings not only pave the way for further researches that are designed to unravel how a metronomic regimen may affect microbial development but also provide a potential reference for optimizing the chemotherapeutic protocols and selecting the suitable regimen in real-world clinical practice.

## Novelty and Impact

Many pharmaceutical agents influence the composition of intestinal microbiota, while gut microorganisms may also modulate the efficacy and the toxicity of drugs. Our results in HER2-negative metastatic breast cancer patients suggested that different dose-intensity regimens of the same chemotherapeutic agent could make a difference in the diversity, composition, and functional structure of gut microbiota, and the presence of specific bacterial species may act as microbial markers associated with drug resistance monitoring and prognostic evaluation.

## Data Availability Statement

The datasets presented in this study can be found in online repositories. The names of the repository/repositories and accession number(s) can be found below: Sequence Read Archive (https://www.ncbi.nlm.nih.gov/sra) under BioProject PRJNA630839.

## Ethics Statement

The studies involving human participants were reviewed and approved by the Institutional Review Board of China National Cancer Center. The patients/participants provided their written informed consent to participate in this study. Written informed consent was obtained from the individual(s) for the publication of any potentially identifiable images or data included in this article.

## Author Contributions

FM and BX conceived the study. XG, XS, CL, LL, FL, SL, ZY, and BL collected and analyzed the data. XG wrote the whole manuscript. All authors edited the manuscript. Besides that, all authors contributed to the article and approved the submitted version.

## Conflict of Interest

The authors declare that the research was conducted in the absence of any commercial or financial relationships that could be construed as a potential conflict of interest.

## Supplementary Material

Table S1 Bacterial operational taxonomic units detected in the metronomic group and the routine group.

Table S2 Multivariate analysis of the clinicopathological characteristics and the gut microbiota for predicting progression-free survival.
